# Supplementary material for: Multi‐dataset identification of innovative feature genes and molecular mechanisms in keratoconus
Source: J Cell Mol Med. 2024 Sep 19;28(18):e70079. doi: 10.1111/jcmm.70079 (PMC11412914; doi:10.1111/jcmm.70079)
Supplement: Supplementary file 2 — Material S2. [file JCMM-28-e70079-s002.zip › 7.GSEA analysis/6.SNORA74B/KEGG_PRION_DISEASES.html]

Details for gene set KEGG\_PRION\_DISEASES[GSEA]

|  || Dataset | SNORA74B.group.cls#h\_versus\_l |
| Phenotype | group.cls#h\_versus\_l |
| Upregulated in class | l |
| GeneSet | KEGG\_PRION\_DISEASES |
| Enrichment Score (ES) | -0.499321 |
| Normalized Enrichment Score (NES) | -1.4380124 |
| Nominal p-value | 0.048543688 |
| FDR q-value | 0.32148767 |
| FWER p-Value | 0.866 |
Table: GSEA Results Summary

  

Fig 1: Enrichment plot: KEGG\_PRION\_DISEASES      
 Profile of the Running ES Score & Positions of GeneSet Members on the Rank Ordered List

  

| SYMBOL | TITLE | RANK IN GENE LIST | RANK METRIC SCORE | RUNNING ES | CORE ENRICHMENT || 1 | ELK1 | na | 2088 | 0.363 | 0.0007 | No |
| 2 | C9 | na | 2720 | 0.318 | 0.0358 | No |
| 3 | C6 | na | 7702 | 0.143 | -0.0838 | No |
| 4 | C8A | na | 8758 | 0.119 | -0.0943 | No |
| 5 | C8G | na | 13060 | 0.046 | -0.2105 | No |
| 6 | C7 | na | 13878 | 0.037 | -0.2278 | No |
| 7 | MAP2K2 | na | 14904 | 0.025 | -0.2531 | No |
| 8 | HSPA1A | na | 16403 | 0.010 | -0.2946 | No |
| 9 | MAPK3 | na | 16753 | 0.007 | -0.3035 | No |
| 10 | C8B | na | 17141 | 0.003 | -0.3141 | No |
| 11 | NOTCH1 | na | 17223 | 0.002 | -0.3160 | No |
| 12 | BAX | na | 18953 | -0.016 | -0.3631 | No |
| 13 | C1QC | na | 20504 | -0.034 | -0.4020 | No |
| 14 | MAPK1 | na | 20606 | -0.035 | -0.3990 | No |
| 15 | PRKX | na | 21076 | -0.041 | -0.4057 | No |
| 16 | SOD1 | na | 21117 | -0.041 | -0.3999 | No |
| 17 | STIP1 | na | 22484 | -0.058 | -0.4296 | No |
| 18 | PRKACA | na | 22659 | -0.060 | -0.4245 | No |
| 19 | EGR1 | na | 22915 | -0.064 | -0.4212 | No |
| 20 | HSPA5 | na | 25096 | -0.100 | -0.4672 | No |
| 21 | PRNP | na | 26089 | -0.119 | -0.4760 | Yes |
| 22 | IL1A | na | 26246 | -0.122 | -0.4601 | Yes |
| 23 | C1QA | na | 26646 | -0.131 | -0.4496 | Yes |
| 24 | LAMC1 | na | 27122 | -0.142 | -0.4395 | Yes |
| 25 | NCAM2 | na | 28843 | -0.190 | -0.4573 | Yes |
| 26 | IL1B | na | 30302 | -0.237 | -0.4597 | Yes |
| 27 | MAP2K1 | na | 31129 | -0.275 | -0.4375 | Yes |
| 28 | IL6 | na | 32098 | -0.328 | -0.4105 | Yes |
| 29 | CCL5 | na | 32237 | -0.337 | -0.3581 | Yes |
| 30 | PRKACB | na | 32349 | -0.344 | -0.3037 | Yes |
| 31 | C5 | na | 32411 | -0.348 | -0.2472 | Yes |
| 32 | PRKACG | na | 32698 | -0.368 | -0.1938 | Yes |
| 33 | FYN | na | 33540 | -0.454 | -0.1420 | Yes |
| 34 | C1QB | na | 33797 | -0.494 | -0.0667 | Yes |
| 35 | NCAM1 | na | 34105 | -0.558 | 0.0180 | Yes |
Table: GSEA details [plain text format]

  

Fig 2: KEGG\_PRION\_DISEASES      
 Blue-Pink O' Gram in the Space of the Analyzed GeneSet

  

Fig 3: KEGG\_PRION\_DISEASES: Random ES distribution      
 Gene set null distribution of ES for **KEGG\_PRION\_DISEASES**
